# Infant feeding practice and associated factors of HIV positive mothers attending prevention of mother to child transmission and antiretroviral therapy clinics in Gondar Town health institutions, Northwest Ethiopia

**DOI:** 10.1186/1471-2458-12-240

**Published:** 2012-03-26

**Authors:** Dagnachew Muluye, Desalegn Woldeyohannes, Mucheye Gizachew, Moges Tiruneh

**Affiliations:** 1 Department of Microbiology, Immunology and Parasitology, College of Medicine and Health Sciences, University of Gondar, Gondar, Ethiopia

## Abstract

**Background:**

It has been estimated that 430,000 children under 15 years of age were newly infected with HIV in 2008, and more than 71% are living in sub-Saharan Africa. In the absence of intervention to prevent mother-to-child transmission, 30-45% of infants born to HIV-positive mothers in developing countries become infected during pregnancy, delivery and breastfeeding. The aim of this study was to assess infant feeding practice and associated factors of HIV positive mothers attending prevention of mother to child transmission and antiretroviral therapy clinics of Northwest Ethiopia.

**Methods:**

Institution based cross sectional study was conducted from January to May 2011 among all HIV positive mothers with less than two years old child attending prevention of mother to child transmission and antiretroviral therapy clinics in Gondar Town health institutions. A structured pre-tested questionnaire using interview technique was used for data collection. The data was entered and analyzed using SPSS version 16 statistical package.

**Results:**

A total of 209 HIV positive mothers were included in the study. Of these, 187 (89.5%) had followed the recommended way of infant feeding practice while significant percentage (10.5%) had practiced mixed breast feeding. In multivariate analysis, disclosure of HIV status with their spouse, insufficient breast milk and occupational status were found to be independently associated (p-value of < 0.05) with recommended infant feeding practice. Lack of resource, stigma of HIV/AIDS, and husband opposition were also obtained as factors that influenced choice of infant feeding options by respondents.

**Conclusions:**

Higher proportion of respondents used the recommended way of infant feeding practice by WHO as well as by Ethiopian Ministry of Health. However, mixed feeding in the first 6 months of age, an undesirable practice in infant feeding, were reported in this study. Infant feeding education that is aligned to national policy should be strengthened in primary health care, particularly in situations where prevention of mother to child transmission of HIV is prioritized.

## Background

Approximately 430,000 children under 15 years of age were newly infected with Human Immunodeficiency Virus (HIV) in 2008, and more than 71% are living in sub-Saharan Africa [1]. Without intervention to prevent mother-to-child transmission, 30-45% of infants born to HIV-positive mothers in developing countries become infected during pregnancy, delivery and breastfeeding [2]. The availability of anti-retroviral therapy (ART) during the last trimester of pregnancy and delivery through prevention of mother to child transmission (PMTCT) program reduces transmission of HIV during pregnancy, labor and delivery from 10% to 20% [3], but it does not solve the problem of infant feeding which is responsible for as much as 5-20% of infections [2]. In sub-Saharan Africa, mother-to-child transmission (MTCT) of HIV is responsible for about 90% of HIV infections in children, and about half of these pediatric infections are thought to have been acquired through breastfeeding [4].

The Joint United Nations Program on HIV/AIDS (UNAIDS) and two of its partners (UNICEF and WHO) recommend that HIV-infected mothers should avoid breastfeeding only when replacement feeding is affordable, feasible, acceptable, sustainable, and safe (AFASS). Non breast-fed children born to HIV positive women are at less risk of illness and death only if they can be ensured uninterrupted access to nutritionally adequate breast milk substitutes that are safely prepared [5]. Exclusive replacement feeding (ERF) can reduce HIV-transmission, but is also associated with morbidity related to diarrhoea and respiratory infections. Exclusive replacement (formula) feeding is the most widely used and effective method to prevent MTCT of HIV-1 through breastfeeding in resource-rich settings and is recommended in situations in which this is AFASS [6-8].

Exclusive breast feeding by HIV-infected mother, when compared to partial breastfeeding or mixed breast feeding (MBF), has been shown to be associated with a reduced risk of transmission in the early months of postpartum [9], and may confer a continued lower risk of transmission in babies continuing to breastfeed from 6-18 months [10,11]. According to the recent guidelines of WHO, when ARVs are not available, mothers should be counseled to exclusively breastfeed in the first six months of life and continue breastfeeding thereafter unless environmental and social circumstances are safe for, and supportive of, replacement feeding [12]. Mixed Breast feeding during the first month of life and breast feeding duration are strong determinants of HIV transmission [13]. The fact that EBF carries a significantly lower risk (almost half the risk) of MTCT of HIV than mixed feeding is not surprising because the beneficial immune factors of breast milk are probably counteracted by the damage to the infant's gut wall by contaminants or allergens in mixed feeds [14].

In a cross sectional study conducted in Addis Ababa, Ethiopia, the proportion of HIV positive mothers who fed their infants ERF, EBF and MBF were 46.8%, 30.6%, and 15.3%, respectively. The predictors for choosing ERF were mode of delivery, household income and disclosure of HIV status to spouse. The predictor for EBF was mode of delivery while for MBF, disclosure of HIV status to spouse, parental infant feeding attitude and infant illnesses were the predictors. Furthermore, sticking to mothers' informed safer feeding options is challenged by some social factors [15].

With the rising prevalence rate of HIV/AIDS, over 200,000 children under-five are HIV positive in Ethiopia. The risk of transmission varies with the duration of breastfeeding, but it is estimated to be about 10-20% for those breastfed for two years [16]. However there is a dearth of literature in the study area particularly in Gondar referral teaching hospital, which is supposed to serve around four millions people coming from and neighboring regions. Therefore, this study has assessed the infant feeding practice of HIV positive mothers including the determinants, and provide evidence-based information that can be used by concerned bodies so that mother to child transmission of HIV virus through breast feeding can be decreased.

## Methods

### Study design, area and period

A cross-sectional study was conducted in Gondar Town PMTCT and ART health institutions, Northwest Ethiopia from January to May, 2011. Gondar town is located in North Gondar zone of Amhara regional state, Northwest Ethiopia 737 km far from the capital, Addis Ababa. Gondar has a total population of 206, 987 and out of these 52.6% are females and 47.4% are males.

### Target population and study population

All HIV positive mothers with less than two years old child attending PMTCT and ART clinics in Gondar Town governmental health institutions.

### Sampling procedures

All (209) HIV positive mothers with less than two years old child attending PMTCT and ART clinics in Gondar Town governmental health institutions were recruited. Those who fulfilled the inclusion criteria were interviewed during their visit to PMTCT and ART clinics.

### Operational definitions

□ Exclusive breast feeding - Giving the infant no other food or drink, not even water, apart from breast milk (including expressed breast milk), with the exception of drops or syrups consisting of vitamins, mineral supplements or prescribed medicines up to six months.

□ Mixed breast feeding - Breastfeeding with the addition of fluids, solid feeds and non-human milks in the first 6 months of age.

□ Exclusive replacement feeding - The process of feeding a child who is not receiving breast milk with a diet that provides all the nutrients the child needs, until the child is fully fed on family foods.

□ Recommended infant feeding practice: those who practiced either exclusive breast feeding or exclusive replacement feeding.

□ Not Recommended: those who practiced mixed breast feeding.

□ Kebele: lowest administrative unit.

### Data collection procedures

Data from all the participants were collected by 4 data collectors and the investigator. The data collectors were selected by their previous experience and education level holding bachelor degree in nursing and one with diploma nurse. They were provided with training on the process and objective of data collection by the investigator. Data was collected using pretested structured questionnaire based interview which includes full details of infant feeding practice and factors likely to influence infant feeding practice of HIV positive mothers. The interview was conducted during their follow up visit of the health institutions. Appropriate modifications were made after analyzing the pre-test result before the actual data collection. The questionnaire was first prepared in English then translated to local language (Amharic).

### Data analysis

The returned questionnaires were checked for completeness, cleaned manually and entered and analyzed using SPSS version 16 statistical package. Data were summarized using frequency tables and pie charts. Backward Stepwise logistic regression model was fitted to identify different determinants of infant feeding practice. Standard techniques for model checking, including the Hosmer-Lemeshow goodness of fit test, were carried out to determine the adequacy of the regression model. Statistical significance was inferred at P-value < 0.05.

### Ethical considerations

This study was approved by Ethical review committee of University of Gondar, College of Medicine and Health sciences, Department of Microbiology, Immunology and Parasitology. Informed consent was obtained from each mother prior to study participation. In order to keep confidentiality of any information provided by study subjects, the data collection procedure were anonymous.

## Results

### Socio-demographic characteristics

A total of 209 HIV positive mothers participated in the study. Of these, 200 (95.7%) were from urban and the rest from rural kebeles. The age of mothers ranges from 18-45 years with an average and standard deviation (SD) of 28.6 and 4.34, respectively. Among the study subjects 189 (90.4%) were Orthodox Christian while 20 (9.6%) were Muslim. The majority of the participants 165 (78.9%) were married and 100 (47.8%) had no formal education. Out of the total study participants 121 (57.9%) were housewives followed by 60 (28.7%) daily laborers. Majority of the participants 161 (77.2%) had a monthly income of less than or equal to 500 Ethiopian birr. The mean age of the infants was 9 months (SD = 5.8) and 54.5% were males [Table 1].

**Table 1:** Socio demographic characteristics of the study subjects attending PMTCT and ART clinics in Gondar town health institutions from January to May, 2011

Characteristics		Frequency	(%)
**Age of mother**	18-24	34	16.3
	
	25-29	88	42.1
	
	30-34	58	27.8
	
	35+	29	13.8

**Religion**	Orthodox	189	90.4
	
	Muslim	20	9.6

**Residence**	Urban	200	95.7
	
	Rural	9	4.3

**Marital status**	Married	165	78.9
	
	Single	44	21.1

**Educational status**	No formal education	100	47.8
	
	Primary education	56	26.8
	
	Secondary and above	53	25.4

**Occupational status**	Housewife	121	57.9
	
	Daily laborer	60	28.7
	
	Government worker	14	6.7
	
	Private/merchant, farmers, others	14	6.7

**Income (in Ethiopian birr)**	< = 200	67	32.0
	
	201-300	41	19.6
	
	301-500	53	25.4
	
	> 501	48	23.0

### Infant feeding practice and other HIV related concerns

The majority 189 (90.4%) of the respondents gave birth between thirty eight and forty eight weeks of gestational age (term), 18 (8.6%) of the respondents had post term birth, and only 2(1%) had pre-term birth. Among these 186 (89%) gave birth at health institutions, 13 (6.2%) gave birth at home with birth attendants and the rest gave birth at home without birth attendants. From the total respondents, 167 (79.9%), 25 (12%), 15 (7.1%) and 2 (1%) had spontaneous vaginal delivery, Cesarean section delivery, Episiotomy and instrumental delivery, respectively.

Among the respondents 138 (66%) and 71 (34%) are on ART and not on ART, respectively. Of the 71 respondents who were not on ART, 46 (64.8%) had taken Neverapin drug starting from 28 weeks of gestational age, 3 (4.2%) had taken a week before labor, 9 (12.7%) had taken during labor and 13 (18.3%) had never taken Neverapin drug.

One hundred eighty three (87.6%) of the respondents had free discussion or disclosure about their HIV status with their spouse while 26 (12.4%) had not disclosed their HIV status with their spouse. Of the participants 137 (65.6%) had free discussion or disclosure of their HIV status with their family and 72 (34.4%) had not disclosed. One hundred twenty two (58.4%), 116 (55.5%), 22 (10.5%), 8 (3.8%), and 6 (2.9%) of the respondents stated that they had got the information about infant feeding from nurses, counselors, mass media, their families and their friends, respectively. Only 4 (1.9%) of the respondents said that they had never got advice on infant feeding at all. Out of the total respondents, 193 (92.3%), 8 (3.8), 4 (1.9%), 3 (1.4%) and 1 (%) knew that MTCT of HIV virus can occur during pregnancy, delivery and breast milk feeding; during delivery and breast milk feeding; only during delivery; only during breast milk feeding and only during pregnancy and delivery, respectively.

Eighty two (39.2%) of the respondents considered that EBF is the only infant feeding option while 64 (30.6%) of the respondents considered that EBF, ERF, heat treated milk and wet nursing are infant feeding options of HIV positive mothers. Twenty nine (13.9%) of the respondents said that EBF, ERF and heat treated milk are infant feeding options of HIV positive mothers while 9 (4.3%) of the respondents said that ERF is the only infant feeding option.

**Figure 1:**
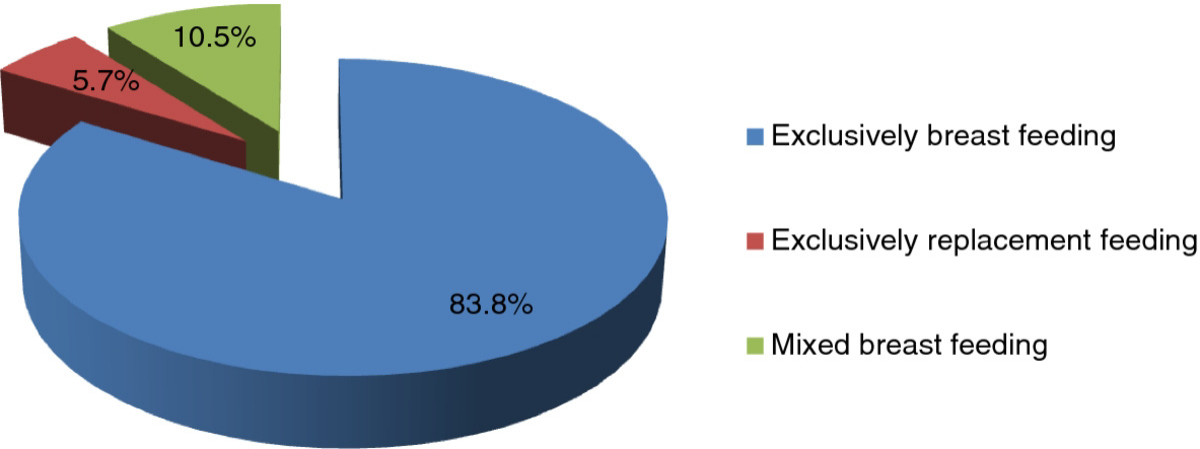
**Percentage of infant feeding practice of HIV positive mothers attending PMTCT and ART clinics in Gondar town health institutions from January to May, 2011**.

One hundred eighty-seven (89.5%) of the study participants had followed EBF and ERF practice while significant percentage (10.5%) of the study participants had practiced mixed feeding [See Figure 1]. One hundred seventy two (82.3%) of the respondents had initiated breast feeding within an hour of delivery and 37 (17.7%) of the respondents had not initiated breast feeding within one hour of delivery. One hundred seventy-five (83.8%) of the study subjects had practiced EBF for their infants and 12 (5.7%) had practiced ERF and no one was reported to practice heat treated milk and wet nursing. The majority of the respondents 138 (66%) use cup for infant feeding while others 57 (27.3%) and 14 (6.7%) use bottle feeding and spoon feeding, respectively. Sixty-seven (32.1%), 27 (12.9%), 24 (11.5%) and 20 (9.6%) of the respondents said that availability of supply, stigma of HIV/AIDS, insufficient breast milk and husband opposition had affected their infant feeding option, respectively.

### Factors affecting infant feeding practice

In logistic regression [see Table 2], place of delivery and disclosure of HIV status with their spouse were found to have association (p-value < 0.05) for those who practiced recommended way of infant feeding practice (EBF and ERF) with COR = 4.98 (95% CI = 1.77-14.01), COR = 3.13 (95% CI = 1.09-8.91) COR = 29.36 (95% CI = 2.91-296.4). In multivariate analysis, disclosure of HIV status with their spouse, insufficient breast milk and occupational status were found to be independently associated (p-value < 0.05) with recommended way of infant feeding practice (EBF and ERF) AOR = 7.7 (95%CI = 1.1-53.97), AOR = 0.14 (95%CI = 0.03-0.65), AOR = 14.63 (95%CI = 1.36-156.40). Mothers who disclose their HIV status to spouse were 7.7 times more likely to have recommended way of infant feeding practice and those daily laborers were 14.6 times more likely to have recommended way of infant feeding practice than private/merchants. Presence of insufficient breast milk was 85.7% times less likely to have recommended way of infant feeding practice.

**Table 2:** Impact of selected socio-demographic and other characteristics on infant feeding practice of HIV positive mothers attending PMTCT and ART clinics in Gondar town health institutions from January to May, 2011

Variables	Infant Feeding Practice		OR (95%CI)		P value
		
	Recommended (%)	Not recommended (%)	Crude	Adjusted	
**Age**					
18-24	28(82.4)	6(17.6)	1	1	
25-29	82(93.2)	6(6.8)	.34(.10-1.14)	.22(.04-1.18)	
30-34	50(86.2)	8(13.8)	.74(.23-2.37)	.85(.16-4.28)	
35+	27(93.1)	2(6.9)	.34 (.06-1.86)	.40(.05-3.14)	

**Residence**					
Urban	179(89.5)	21(10.5)	1.06(.12-8.94)	.99(.07-12.66)	
Rural	8(88.9)	1(11.1)	1	1	

**Religion**					
Orthodox Christian	170(89.9)	19(10.1)	1.57(.42-5.88)	2.50(.43-14.49)	
Muslim	17(85.0)	3(15.0)	1	1	

**Marital status**					
Married	149(90.3)	16(9.7)	1.47(.53-4.01)	.79(.13-4.75)	
Single	38(86.4)	6(13.6)	1	1	

**Educational status**					
No formal education	89(89.0)	11(11.0)	1.03(.35-2.96)	3.08(.60-15.69)	
Primary education	51(91.1)	5(8.9)	1.30(.373-4.55)	2.80(.483-16.23)	
Secondary and above	47(88.7)	6(11.3)	1	1	

**Occupational status**					0.026
House wife	108(89.3)	13(10.7)	2.26(.55-9.19)	4.64(.66-32.26)	
Daily laborer	54(90.0)	6(10.0)	2.45(.53-11.3)	14.63(1.36-156.4)*	
Government/Private worker/merchants	25(89.3)	3(10.7)	1	1	

**Monthly income**					
< = 200	55(82.1)	12(17.9)	.41(.12-1.38)	.53(.11-2.58)	
201-300	38(92.7)	3(7.3)	1.15(.24-5.47)	1.60(.23-10.95)	
301-500	50(94.3)	3(5.7)	1.51(.32-7.14)	3.73(.52-26.32)	
> 501	44(91.7)	4(8.3)	1	1	

**Place of delivery**					
Health institution	171(91.9)	15(8.1)	4.98(1.77-14.01)*	2.26(.47-10.88)	
At home	16(69.6)	7(30.4)	1	1	

**Disclosure of HIV status to spouse**					0.039
yes	167(91.2)	16(8.8)	3.13(1.09-8.91) *	7.74(1.11-53.97) *	
no	20(76.9.5)	6(23.1)	1	1	

**Disclosure of HIV status to family**					
yes	124(90.5)	13(9.5)	1.36(.55-3.36)	.38(.08-1.72)	
no	63(87.5)	9(12.5)	1	1	

**On ART**					
Yes	123(89.1)	15(10.9)	.89(.34-2.31)	.93(.28-3.07)	
no	64(90.1)	7(9.9)	1	1	

**Insufficient milk**					0.013
Yes	19(79.2)	5(20.8)	.38(.12-1.16)	.14(.03-.65)*	
No	168(90.8)	17(9.2)	1	1	

Note: 1- reference group

* Statistically significant (p-value < 0.05)

## Discussion

In this study, the proportion of mothers practicing EBF (83.7%) for the first 6 months of age was comparatively higher than the findings reported from Nigeria (68.3%), Uganda (24%), India (44%) and South Africa (35.6%) [17-20] and the proportions of mothers practicing ERF (5.7%) was lower than reported from South Africa (50%), India (44%), and Nigeria (31.7%) [17-19]. This might be due to the culture of feeding habit of the Ethiopian mothers to their children than giving replacement feeding as well as the availability of resources to practice ERF. The proportion of mothers practicing EBF was also comparatively higher than what was reported from Addis Ababa, Ethiopia (30.6%) [15]. The Ethiopian Ministry of Health guideline on infant feeding recommendations of HIV exposed infants recommends EBF for the first 6 months and introducing complementary feeding at 6 months and continues breastfeeding until 12-18 months [21]. The difference may be also explained by the fact that in both the South African and Nigerian studies, infant formula was supplied free of charge unlike in the present study. Deep-rooted family and community norms make it difficult for mothers in Ethiopia as in most developing countries to choose ERF. In fact, choosing to use replacement feed is equivalent to announcing their HIV status, and the consequences of this are far reaching and could involve violence and divorce [22]. About 87% of the mothers had disclosed their HIV status to their spouses, and 65.6% of the mothers had disclosed their status to their family members. This number is found to be higher than what was reported in Nigeria (50%) [19].

In multivariate analysis, disclosure of HIV status with their spouse, insufficient breast milk and occupational status were found to be independently associated with the recommended way of infant feeding practice. Mothers who disclose their HIV status to spouse were 7.7 times more likely to have the recommended way of infant feeding practice. Disclosure of HIV status greatly influenced infant feeding options of HIV positive mothers when the partner was aware of the HIV status of the mother and involved in the decision [23]. Having disclosed their status might have psychological benefits as they do not have to hide while formula feeding. Those daily laborers were 14.6 times more likely to have recommended way of infant feeding practice than government/private workers/merchants. Work overload may dispose merchants to practice MBF unconsciously.

The proportion of mothers practicing MBF (10.5%) for the first 6 months of age was almost in line with studies conducted in Addis Ababa, Ethiopia (15.3%) and South Africa (12.4%) [15,17], but lower than from India (29%) [18] and it was higher than what was reported from Cameroon (4.3%) [24]. By tradition, mothers may consider breast milk is not enough for child growth and intend to use mixed feeding even though they have been informed. Mixed breast feeding has been shown to damage the intestinal lining of the gut in infants [14], leading to an increased risk of HIV transmission through breast milk [9,14]. In addition to MBF, duration of breast feeding is also strong determinant for HIV transmission through breast milk [13]. According to a study conducted in Zambia, early abrupt cessation of breast-feeding by HIV-infected women in a low-resource setting does not improve the rate of HIV-free survival among children born to HIV-infected mothers [25]. The recent guidelines of WHO stated that when ARVs are not available, mothers should be counseled to exclusively breastfeed in the first six months of life and continue breastfeeding thereafter unless environmental and social circumstances are safe for, and supportive of, replacement feeding [12]. Presence of insufficient breast milk was 85.7% times less likely to have recommended way of infant feeding practice. Due to the insufficiency of breast milk mothers might goes to mixed feeding.

In the present study, 193 (92.3%) of women knew that mother to child transmission of HIV virus can occur during pregnancy, delivery and breast milk feeding which is a higher percentage compared to the findings (70%) from a study done in South Africa [17]. This might be achieved due to the accessibility of mother support groups by preparing coffee ceremony for mothers in two weeks interval which is organized by NGO. However, without fostered counseling including information about the risks and benefits of breastfeeding, HIV-positive women may have made suboptimal infant feeding decisions (e.g., mixed feeding, underfeeding) based only on the knowledge that HIV can be transmitted by breastfeeding. In an effort to prevent HIV transmission to their infants, women may be unintentionally endangering their infants' health. This emphasizes the need for offering clear information about postpartum HIV transmission to all women seeking antenatal care.

Availability of supply, stigma of HIV/AIDS, insufficient breast milk and husband opposition were factors that influenced choice of infant feeding options by respondents. Higher percentage (13%) of study participants complained that stigma of HIV/AIDS status affect their infant feeding option although the percentage is lower than studies conducted in Nigeria (44%) [23,26]. This might be due to the cultural difference in accepting HIV/AIDS in the people of each country.

The study findings are limited in terms of overall generalization due to the small study sample size and it was health institution based. There is a possibility that study participants who received counseling on recommended way of infant feeding practice may simply answer questions accurately. This bias may underestimate the proportion of mixed feeding practice. Maternal since-birth recall of feeding patterns was also used which has its own limitations of long recall. Despite these limitations, we believe that our study findings provide essential input on infant feeding decisions.

## Conclusions

The present study showed and identified that higher proportion (89.5%) of the respondents used the recommended way of infant feeding practice. Mixed feeding (10.5%), an undesirable practice in infant feeding, in the first 6 months of age were also reported. Major determinants of infant feeding practice were found to be disclosure of HIV status with their spouse, insufficient breast milk and occupational status. However, further advanced clinical trial studies have to be conducted in order to see the effect of different infant feeding practice for maternal viral transmission through breast milk.

## Abbreviations

AFASS: Affordable, Feasible, Acceptable, Sustainable, and Safe; AIDS: Acquired Immune Deficiency Syndrome; ART: Anti Retroviral Therapy; EBF: Exclusive Breast Feeding; ERF: Exclusive Replacement Feeding; HIV: Human Immunodeficiency Virus; MBF: Mixed Breast Feeding; MTCT: Mother to Child Transmission; NGOs: Non Governmental Organizations; OR: Odds Ratio; PMTCT: Prevention of Mother to Child Transmission; SPSS: Statistical Packages for Social Sciences; UNAIDS: United Nations Program on HIV/AIDS; WHO: World Health Organization.

## Competing interests

The authors declare that they have no competing interests.

## Authors' contributions

DM: initiation of the study, design, implementation, analysis and writing. DW: design, implementation, analysis and writing. MG: implementation and co-writing. MT: design, implementation of the study and co-writing. All authors read and approved the final manuscript.

## Pre-publication history

The pre-publication history for this paper can be accessed here:

http://www.biomedcentral.com/1471-2458/12/240/prepub

## References

[B1] UNAIDS Global summary of the AIDS epidemic. AIDS epidemic update 2008

[B2] De Cock KM Fowler MG Mercier E de Vincenzi I Saba J Hoff E Alnwick DJ Rogers M Shaffer N Prevention of mother-to-child HIV transmission in resource-poor countries: translating research into policy and practice JAMA 2000 283 1175 1182 10.1001/jama.283.9.1175 10703780

[B3] Guay L Musoke P Fleming T Bagenda D Allen M Nakabiito C Sherman J Bakaki P Ducar C Deseyve M Emel L Mirochnick M Fowler MG Mofenson L Miotti P Dransfield K Bray D Mmiro F Jackson JB Intrapartum and neonatal single-dose nevirapine compared with zidovudine for prevention of mother-to-child transmission of HIV-1 in Kampala, Uganda: HIVNET 012 randomized trial Lancet 1999 354 795 802 10485720 10.1016/S0140-6736(99)80008-7

[B4] Newell M Current issues in the prevention of mother-to-child transmission of HIV-1 infection Trans R Soc Tropical Medicine and Hygiene 2006 100 1 5 10.1016/j.trstmh.2005.05.012 16214193

[B5] UNAIDS/WHO HIV and infant feeding: A Policy Statement developed collaboratively by UNAIDS, UNICEF and WHO 1998 UNAIDS/WHO, Geneva, Switzerland 10453706

[B6] Nduati R John G Mbori D Richardson B Overbaugh J Mwatha A Ndinya-Achola J Bwayo J Onyango FE Hughes J Kreiss J Effect of breastfeeding and formula feeding on transmission of HIV-1: a randomized clinical trial JAMA 2000 283 1167 1174 10.1001/jama.283.9.1167 10703779

[B7] Embree JE Njenga S Datta P Nagelkerke NJ Ndinya-Achola JO Mohammed Z Ramdahin S Bwayo JJ Plummer FA Risk factors for postnatal mother-child transmission of HIV-1 AIDS 2000 14 2535 2541 10.1097/00002030-200011100-00016 11101065

[B8] Athena Kourtis P Denise Jamieson J Isabelle de Vincenzi Allan Taylor Michael Thigpen C Halima Dao Timothy Farley Mary Glenn Fowler Prevention of human immunodeficiency virus-1 transmission to the infant through breastfeeding: new developments American Journal of Obstetrics & Gynecology 2007 197 113 122 17825642 10.1016/j.ajog.2007.03.003

[B9] Coovadia Hoosen M Rollins Nigel C Bland Ruth M Kirsty Little Anna Coutsoudis Bennish Michael L Marie-Louise Newell Mother-to-child transmission of HIV-1 infection during exclusive breastfeeding in the first 6 months of life: an intervention cohort study Lancet 2007 369 1107 1116 10.1016/S0140-6736(07)60283-9 17398310

[B10] Coutsoudis A Pillay K Spooner E Kuhn L Coovadia HM Influence of infant-feeding patterns on early mother-to-child transmission of HIV-1 in Durban, South Africa: a prospective cohort study South African Vitamin A Study Group. Lancet 1999 354 471 476 10.1016/s0140-6736(99)01101-0 10465172

[B11] Iliff P Piwoz E Tavengwa N Zunguza CD Marinda ET Nathoo KJ Moulton LH Ward BJ Humphrey JH Early exclusive breastfeeding reduces the risk of postnatal HIV-1 transmission and increases HIV-free survival AIDS 2005 19 699 708 10.1097/01.aids.0000166093.16446.c9 15821396

[B12] WHO, UNICEF, UNFPA, UNAIDS Guidelines on HIV and Infant Feeding 2010: Principles and recommendations for infant feeding in the context of HIV and a summary of evidence 2010 24501786

[B13] Becquet R Bland R Leroy V Rollins NC Ekouevi DK Coutsoudis A Dabis F Coovadia HM Salamon R Newell M Duration. Pattern of Breastfeeding and Postnatal Transmission of HIV: Pooled Analysis of Individual Data from West and South African Cohorts PLoS ONE 2009 4 e7397 10.1371/journal.pone.0007397 19834601 PMC2759081

[B14] Renaud Becquet Ekouevi Didier K Hervé Menan Clarisse Amani-Bosse Laurence Bequet Ida Viho François Dabis Marguerite Timite-Konan Valériane Leroy Early mixed feeding and breastfeeding beyond 6 months increase the risk of postnatal HIV transmission Preventive Medicine 2008 47 27 33 10.1016/j.ypmed.2007.11.014 18190955

[B15] Maru Y Haidar J Infant feeding practice of HIV positive mothers and its determinants in selected health institutions of Addis Ababa, Ethiopia Ethiopian Journal Health Development 2009 23 107 114

[B16] Ethiopian National Strategy on Infant and Young Child Feeding 2004

[B17] Rendani L Karl P Motlatso G Khanyisa Phaweni Infant-feeding practices and associated factors of HIV-positive mothers at Gert Sibande, South Africa Acta Pædiatrica 2011 100 538 542 21062356 10.1111/j.1651-2227.2010.02080.x

[B18] Suryavanshi N Jonnalagadda S Erande AS Sastry J Pisal H Bharucha KE Shrotri A Bulakh PM Phadke MA Bollinger RC Shankar AV Infant feeding practices of HIV positive mothers in India Journal of Nutrition 2003 133 1326 1331 12730418 10.1093/jn/133.5.1326

[B19] Adejuyigbe E Orji E Onayade A Makinde N Anyabolu H Infant feeding intentions and practices of HIV-positive mothers in Southwestern Nigeria Journal of Human Lactation 2008 24 303 310 10.1177/0890334408317765 18689717

[B20] Fadnes LT Engebretsen IM Wamani H Semiyaga NB Tylleskär T Tumwine JK Infant feeding among HIV-positive mothers and the general population mothers: comparison of two cross-sectional surveys in Eastern Uganda BMC Public Health 2009 9 124 10.1186/1471-2458-9-124 19422709 PMC2687447

[B21] Guidelines for Prevention of Mother-to-Child Transmission of HIV in Ethiopia Federal HIV/AIDS Prevention and Control Office, Federal Ministry of Health 2007

[B22] Adejuyigbe E Odebiyi A Parental HIV serodiscordance: implications for the care of the HIV seropositive child in a poor-resource setting AIDS Care 2006 18 537 543 10.1080/13548500500228698 16831779

[B23] Mohammed A Shehu AU Aliyu AA Zoaka AI Infant feeding options, practices and determinants of HIV-positive mothers in Abuja, Nigeria Niger Medical Journal 2010 51 14 17

[B24] Njom Nlend A Penda I Same Ekobo C Tene G Monny Lobe M Tsague L Macauley I Zekeng L Engozo'o A International Conference on AIDS (15th:2004: Bangkok, Thailand). Factors associated with infant feeding choices of HIV positive mothers in urban areas Cameroon Int Conf AIDS 2004 15 11 16

[B25] Kuhn L Aldrovandi GM Sinkala M Kankasa C Semrau K Mwiya M Kasonde P Scott N Vwalika C Walter J Bulterys M Tsai W Thea DM Effects of Early, Abrupt Weaning on HIV-free Survival of Children in Zambia N Engl J Med 2008 359 130 141 10.1056/NEJMoa073788 18525036 PMC2577610

[B26] Oladokun R Brown B Osinusi K Infant-feeding pattern of HIV-positive women in a prevention of mother-to-child transmission (PMTCT) programme AIDS Care 2010 22 1108 1114 10.1080/09540120903511008 20229369

